# Everyday Life Meaningfulness for the Community-Dwelling Oldest Old During the COVID-19 Pandemic

**DOI:** 10.3389/fpsyg.2021.716428

**Published:** 2021-09-09

**Authors:** Elisa Tiilikainen, Inna Lisko, Eija Kekkonen, Alina Solomon, Tiia Ngandu, Miia Kivipelto, Jenni Kulmala

**Affiliations:** ^1^Department of Social Sciences, University of Eastern Finland, Kuopio, Finland; ^2^Faculty of Sport and Health Sciences and Gerontology Research Center (GEREC), University of Jyväskylä, Jyväskylä, Finland; ^3^Division of Clinical Geriatrics, Center for Alzheimer Research, Department of Neurobiology, Care Sciences and Society, Karolinska Institutet, Stockholm, Sweden; ^4^Institute of Clinical Medicine, School of Medicine, University of Eastern Finland, Kuopio, Finland; ^5^Ageing Epidemiology Research Unit, School of Public Health, Imperial College London, London, United Kingdom; ^6^Population Health Unit, Finnish Institute for Health and Welfare, Helsinki, Finland; ^7^Institute of Public Health and Clinical Nutrition, University of Eastern Finland, Helsinki, Finland; ^8^Faculty of Social Sciences (Health Sciences) and Gerontology Research Center (GEREC), Tampere University, Tampere, Finland

**Keywords:** oldest old, meaningfulness, everyday life, COVID-19, mixed methods

## Abstract

In many countries, the COVID-19 pandemic has led to strong restrictions and changed the everyday lives of older people. In Finland, people aged 70 and over were instructed to stay at home under quarantine-like conditions. Existing studies from other countries have reported increases in negative experiences and symptoms as a result of such restrictions, including psychosocial stress. However, little focus has been given to older people’s experiences of meaningfulness during the pandemic. Using survey and interview data, we ask to what extent have community-dwelling oldest old (80+) experienced meaningfulness during the pandemic, what background factors are associated with meaningfulness and what factors have contributed to everyday life meaningfulness during the pandemic. The data was collected as part of the COVID-19 sub-study of the third follow-up of the Cardiovascular Risk Factors, Aging and Dementia (CAIDE85+) study, a Finnish population-based cohort study carried out in the eastern part of the country. In the quantitative analyses, meaningfulness was assessed as part of the Experiences of Social Inclusion Scale. The association of meaningfulness with different background factors (gender, age, living alone, self-chosen quarantine or physical isolation, self-rated health, physical functioning, and cognitive capacity) was explored with the Chi-square test. The quantitative findings indicate that the majority of the participants experienced meaningfulness during the pandemic. Participants who did not practice any physical isolation measures and participants with higher self-rated health experienced more meaningfulness. There was no evidence for difference in the prevalence of meaningfulness and other background factors. The qualitative data was analyzed using thematic analysis. The findings indicated that factors contributing to meaningfulness in everyday life were social contacts, daily chores and activities, familiar places and seasonal changes. The small sample size does not provide possibilities for generalizing the results into the wider population of older adults. However, the results provide new understanding of the oldest old’s experiences of meaningfulness in everyday life during the global pandemic. The findings may help find ways to support older people’s meaningfulness in challenging times.

## Introduction

The ongoing COVID-19 pandemic and its related restrictions have put older people in a situation in which their possibility to maintain normal daily activities has been compromised. In Finland, the Emergency Powers Act was implemented on March 16, 2020 and was continued until June 16, 2020. The act included decisions such as closure of public services, e.g., libraries and museums; severe limitations of public gatherings; suspension of contact teaching; reduction of non-acute social and health care services; and a ban on visiting care institutions and hospitals. A strong recommendation was given that all older adults aged 70 and over should stay at home and avoid all physical contacts. Many of these restrictions and recommendations have been in force over a year. Although the aim has been to protect older adults and their health during the exceptional times, the consequences and older people’s own experiences related to these restrictions are not fully understood.

In this article we examine community-dwelling oldest old’s (80+) experiences during the COVID-19 pandemic from the perspective of *everyday life meaningfulness*. While the definition of meaningfulness varies across scholars and disciplines, it is often understood as having a sense of purpose in life and experiencing life as significant (e.g., Baumeister, 1991; King et al., 2006; Steger, 2012). According to Baumeister (1991), meaningfulness in life can be understood in terms of four main needs for meaning: purpose, value, sense of efficacy and self-worth. If these needs–fulfillment of goals, justification for actions, feeling that one can reach one’s goals, and positive self-worth–are not met a person is more likely to experience less meaning in life. Moreover, Baumeister and Vohs (2002) propose that having multiple sources of meaning in life protects the individual against meaninglessness highlighting the multidimensional nature of meaningfulness: one can gain purpose and self-worth from different spheres of life, such as family and work. In older ages these spheres are often narrowed due to losses of social relations and roles and decrease in functional capabilities (e.g., Tiilikainen and Seppänen, 2016).

Drawing on the conceptualization by Steger (2012) we understand meaningfulness as a web of connections, understandings and interpretations that help comprehend individual experiences, direct efforts toward desired futures, and provide a sense that life is worthwhile. In further research, Martela and Steger (2016) have highlighted the importance of understanding the difference between meaning in life and the more philosophical question about meaning of life. Within the aim of looking at the subjective experiences of what makes live meaningful, Martela and Steger (2016) have proposed three facets of meaning: coherence, purpose and significance. These include a sense of comprehensibility and making sense of one’s life, sense of core goals, aims and direction in life and a sense of life’s inherent value and having a life worth living. In other words, in order to live a meaningful life, people need to comprehend the world around them, they need to find direction for their actions, and they need to find worth in their lives.

Perceiving life as meaningful plays an important part in well-being at all ages, but particularly in later life (see Jonsén et al., 2014). For the oldest old meaningfulness in life is known to contribute to not only quality in life and mental wellbeing but also physical health (e.g., King et al., 2006; Krause, 2007). In an interview study carried with the oldest old, Jonsén et al. (2014) found that the sense of meaning in life was linked to having a mission to pursue and finding beauty, joy and happiness in life. The sense of meaning also involved being connected with other people and with nature, and seeing oneself as part of a chain of generations. Moreover, a sense of meaningfulness was deeply rooted in the faith of being taken care of from birth to the afterlife. In this article, our focus is on the everyday life components providing a sense of meaningfulness rather than facets of meaningfulness itself. From the perspective of everyday life, meaningfulness provides a sense that one’s daily life matters and has a purpose in this current moment and surrounding social and environmental setting, which for the oldest old is often the home environment.

Both theoretical and empirical research on meaningfulness show that social relations and having the possibility to impact one’s own life play an important role in experiencing life as meaningful (e.g., Ryan and Deci, 2004; Hicks and King, 2009; Heintzelman and King, 2014). Arguably, these factors have at least been somewhat challenged due to the COVID-19 pandemic as many people have avoided physical contact and adjusted their lives governmental restrictions and recommendations. However, as some studies have already shown, older people have reported less pandemic related negative impacts than younger people (Jiang, 2020; Birditt et al., 2021; O’Connor et al., 2021; see also Kivi et al., 2021) and therefore may have been less affected by the pandemic than younger generations. Moreover, studies have found that older people have reached various coping strategies in adapting to the pandemic-related changes and challenges in everyday life (Van Tilburg et al., 2021).

To our knowledge, previous research has not focused on older people’s own views of the everyday life meaningfulness during the pandemic. Yet, some qualitative COVID-19 studies have referred to perspectives that are relevant to meaningfulness (Brooke and Clark, 2020; Portacolone et al., 2021; Whitehead and Torossian, 2021). Brooke and Clark (2020) conducted a follow-up interview study in United Kingdom focusing on older people’s responses to the pandemic, including protective measures, ways of coping and future plans. Their interviewees expressed the need to embrace and live life, as well as the need to be distracted from the pandemic. The findings show that older adults found watching nature and wildlife important, as well as keeping busy with daily chores. In addition, social media was seen to be important in keeping in touch with friends and family. Despite the restrictive measures, some of the interviewed older adults expressed being blessed and fortunate, and many felt that they did not fear the future.

An interview study by Portacolone et al. (2021) found that older adults living alone with cognitive impairment experienced distress, including fear and confusion, as well as extreme isolation and loneliness during the pandemic. Despite their negative experiences, the interviewees reported using a number of coping strategies, such as exercising and outdoor routines, attending religious events and thinking positively about the future. Similarly, Whitehead and Torossian (2021) focused on stressful and joyful factors in their mixed-methods study on the lives of older adults during the pandemic. Their results indicated that stress was related to confinement and/or restrictions, concern for others, isolation and loneliness. Sources of joy were found in family and friend relationships, digital social contacts, and hobbies. Faith, exercise, other forms of self-care and nature were associated with more positive psychological well-being during the pandemic. Similar factors are known to contribute to meaningfulness in later life (Jonsén et al., 2014).

Despite large amount of research on the effects of COVID-19 pandemic, the oldest old’s lived experiences, as well as the positive aspects of the pandemic have received very little attention. In this article, we aim to fill this research gap by giving voice to the oldest old’s own experiences of everyday life meaningfulness during the COVID-19 pandemic. Our specific research questions are:

• To what extent have the community-dwelling oldest old experienced meaningfulness during the COVID-19 pandemic and what background factors are associated with meaningfulness?
• What factors have contributed to maintaining everyday life meaningfulness during the COVID-19 pandemic?

## Materials and Methods

The study utilizes a mixed-methods approach combining quantitative and qualitative data and analysis. The purpose of using two different types of materials and methods is to gain better and more complete understanding of the lived experiences of older people during the COVID-19 pandemic. To this end, we use an explanatory mixed methods design (Ivankova and Creswell, 2009). The different data sets–surveys and telephone interviews–were collected and analyzed in sequence, proceeding from quantitative data to qualitative data. Quantitative data is used to give an overview of the study populations experiences of meaningfulness when answering the first research question. Qualitative findings are used to explain and extend the quantitative results by answering the second research question.

The data is derived from the COVID-19 sub-study of the third follow-up of the Cardiovascular Risk factors, Aging and Dementia (CAIDE85+) study. All the participants lived in Eastern Finland and came originally from five population-based cohorts who were assessed in mid-life in 1972–1992 (Barbera et al., 2020). Altogether 316 invitations were sent for the COVID-19 sub-study (survey and telephone interview). Invitations were sent in mid-July 2020 to all individuals who had participated in the main assessment of the CAIDE85+ study and given their consent for further research contact (*n* = 140), and to individuals who had not yet participated in the CAIDE85+ main assessment (*n* = 176). A round of reminder letters was sent in late August or early September, or in connection with the CAIDE 85+ main assessment. Altogether 211 individuals did not participate in the COVID-19 sub-study (141 individuals declined to participate (information was received by email or phone), 16 individuals were reported deceased by a relative, and 54 individuals were not reached). Reasons for declining to take part in the study included e.g., poor health, feeling tired/not having energy to participate, and a recent participation in the CAIDE85+ main assessment.

Altogether 103 persons provided answers to the survey from the participants (*N* = 105) who had given their consent. In all, 64% of them responded by the end of July, 79 % by the end of September and the rest between September 13th and December 10th. Qualitative telephone interviews were carried out with 15 participants between August and December. All the study participants were community-dwelling. A more detailed description of the participants is presented in Table 1 in the beginning of the “Results” section.

**TABLE 1:** Characteristics of the participants in the CAIDE 85+ Covid-19 sub-study.

	*n* ^*^	All	*n* ^*^	Interviewees
Age, Mean (SD)	103	85.8 (5.3)	15	84.8 (7.3)
Women, %	103	58.7	15	66.7
Living alone, %	96	69.8	15	66.7
Quarantine/physical isolation currently	95		15	
No quarantine or physical isolation	21	22.1	5	33.3
Quarantine, self-imposed	5	5.3	0	0.0
Avoiding physical contacts	69	72.6	10	66.6
Self-rated health	103			
Poor	14	13.6	1	6.7
Average or higher	89	86.4	14	93.3
Cognitive capacity	83		14	
Higher capacity (MMSE score ≥ 25), %	71	85.5	13	92.9
Lower capacity (MMSE score < 25), %	12	14.5	1	7.1
Physical functioning, 500 m walk	101		15	
No walking difficulties, %	61	60.4	9	60.0
At least some walking difficulties, %	29	28.7	3	20.0
Unable to walk 500 m (%)	11	10.9	3	20.0

*The heading “All” refers to all those answering the questionnaire (*n* = 103); the interviewees were a group of 15 people selected from the overall group.*

^*^Presents the number of participants included in each studied variable or response category; For all individuals the *n* ranges between 83 and 103 and for interviewees 14–15, respectively.

*SD = Standard Deviation; MMSE = Mini-Mental State Examination; IQR = Interquartile range; CAIDE85+ = Third follow-up of the Cardiovascular Risk Factors, Aging and Dementia study.*

### Survey and Methods

#### Meaningfulness

Meaningfulness was assessed with three items drawn from the Experiences of Social Inclusion Scale (Finnish Institute for Health and Welfare, 2019) which is still under the validation process (Leemann et al., under review)^1^. The items represent the following statements: (i) “I feel that my life has purpose,” (ii) “I belong to a group or community that is important for me,” and (iii) I feel that what I do everyday is significant.” The given answer options were (i) strongly disagree, (ii) somewhat disagree, (iii) neither agree nor disagree, (iv) somewhat agree, and (v) completely agree. In the analyses, three classes were used: (i) disagree, (ii) neither agree nor disagree, and (iii) agree. The theoretical background for these statements has been drawn from the framework by Martela and Steger (2016) (Leemann et al., under review)^1^ and even though the questions have not been developed to measure meaningfulness in life *per se*, we find that these items reflect well our aim of examining meaningfulness in the context of everyday life.

#### Health-Related Factors

##### Physical functioning

Physical functioning was derived from the question “Are you capable of walking 500 meters without resting?” The answer options were (i) I can without difficulties, (ii) I can, but I have some difficulties, (iii) I can, but it is very difficult, and (iv) I can’t do it. The answer options (ii) and (iii) were combined.

##### Cognition

Cognitive status was derived from the face-to-face main study assessment and assessed with Mini-Mental-State Examination (MMSE) (Folstein et al., 1975). A score of <25 points was set to describe lower cognitive capacity. The used cut point is recommended based on Finnish older population (Hänninen et al., 2010).

##### Health status

Self-rated health was used as an indicator of health status (Jylhä, 2009). The used question was derived from previous data collections of the CAIDE study population: “What do you think about your current health status? Is it very bad, quite bad, average, fairly good or very good?” In the analyses, the classes were (i) poor (very bad or quite bad) and (ii) at least average.

##### Other factors

Age of the participants was categorized into tertiles: <85.5 years, 85.5–88.8 years, >88.8 years. Regarding living situation, the participants reported whether they lived alone or not. The current status of quarantine/physical isolation was asked and the following answer options were given (i) I am not in quarantine or avoiding social contacts, (ii) I am in self-quarantine, (iii) I am in official quarantine, (iv) I am avoiding social contacts, and (v) I am partly avoiding social contacts. The answer options (ii), (iv), and (v) were combined as self-chosen quarantine or physical isolation. None of the participants were in quarantine enforced by law (iii).

#### Statistical Analyses

In this article, the statistical analyses were used to provide an overview of the everyday life meaningfulness of the study participants’ during the pandemic. The survey data were analyzed using IBM SPSS Statistics 27 program. Chi-square test was used in the statistical analyses and differences in perceived meaningfulness were investigated based on age, gender, living status, self-chosen quarantine or physical isolation status, cognition, physical functioning and self-rated health. It was also examined if the whole study population and the interviewees felt differently on meaningfulness. Categorical variables presented earlier were used. A *p* value < 0.05 was considered statistically significant.

### Interview Data and Methods

The qualitative data consists of 15 telephone interviews. The interviewees were selected from survey participants who had given their consent to record the telephone conversation (*N* = 96). As the aim of the study was to gain comprehensive understanding of the COVID-19-related experiences of the oldest old, the interview participants were selected so that they would represent a variety of life situations (e.g., gender and marital status) and experiences (e.g., perceived health and well-being). A research nurse contacted the preselected survey participants by telephone to ask whether they were still willing to discuss their experiences further in a qualitative interview. All 15 contacted persons expressed their interest in and consent to participation in the interviews.

The number of interviewees was chosen based on the resources of the study (one experienced interviewer) and the contents of the interviews. Rather than aiming to recruit as many interviewees as possible, we wanted to gain in-depth understanding of various experiences related to the participants’ everyday life during the pandemic. The interview guide was developed based on the CAIDE85+ COVID-19 survey including similar topics, such as daily life and habits, social interaction, restrictions and quarantine, psychological and health impacts, use of social and health care services and ways of coping with the pandemic. In addition to the general interview guide, some questions were added or modified based on the interviewees’ responses in the survey. Additional and/or complementary questions were asked about issues expressed in the open-ended questions.

The interviews were carried out by telephone in order to ensure that the older people were able to participate in the study during a time of physical distancing. All interviewees were at their private homes during the telephone calls. On some occasions, non-participants were also present, including a spouse, neighbor or a repairman. However, in most cases the interview was carried out in a separate and quiet room, so that the interviewee was able to participate without distractions. In two cases, the interview was divided into two different sessions due to interruptions or problems with the phone line.

As highlighted by Brooke and Clark (2020) in their COVID-19 study, telephone interviews have several benefits including a greater level of anonymity and privacy, removal of visual clues and more balanced distribution of power between the researcher and the participant, which may help the interviewees to feel more comfortable in discussing their personal feelings. At the same time, the lack of visual cues and loss of non-verbal interaction may be regarded as specific challenges when interviewing via telephone. Based on our experiences, both factors–benefits and challenges–were present in the telephone interviews. For the researcher, lack of non-verbal interaction was somewhat challenging during the first interviews, especially when discussing sensitive issues such as loss of a spouse. However, the feedback from the participants was highly positive, and all of them expressed being pleased about the opportunity to discuss their experiences in this way.

The interviews lasted between 45 and 95 min and were audiotaped using a separate dictation machine. Altogether, the audiotaped data included 17 h, transcribed into 321 pages (Verdana font size 12, spacing 1). The excerpts shown in the “Results” section have been translated from Finnish to English by the first author. The final versions were approved by the other authors and a professional translator in order to ensure that the original voices of the participants were not lost (van Nes et al., 2010).

The interview data was analyzed using thematic analysis (Braun and Clarke, 2006). In the first phase of the analysis, the data was read through and a short summary of each interview was made by the first author. These summaries included basic information of the interviewee and notes of the issues discussed in each interview. After this the data was re-read by the first and last Author to obtain a sense of the whole data. During this phase meaningful units, including sentences and paragraphs, were identified and coded with labels, such as “summer cottage” and “walking the dog.” After the initial coding process, codes were collated into themes that were present in all interviews and followed our second research question. The coding process and themes were discussed several times within the research team to ensure the reliability of the analysis. At the final stage, three clear themes were identified: social contacts, daily chores and activities and places and seasonal changes, which were described as meaningful factors in everyday life. Within these main themes, different sub-themes were identified (Table 2).

**TABLE 2:** Themes and sub-themes regarding factors supporting the everyday life meaningfulness during the COVID-19 pandemic.

Social contacts
Telephone and video calls
Face-to-face with family
Friends and acquaintances outdoors
**Daily chores and activities**
Housekeeping and yard work
Reading and knitting
Going for walks
**Places and seasonal changes**
Summer cottage
Nature and outdoors
Spring and summer

### Ethics of the Study

Each participant or their legally acceptable representative signed the informed consent form in the COVID-19 sub-study as well as in the CAIDE85+ main study assessment. Ethics approval for conducting the study was received from The Research Ethics Committee of the Northern Savo Hospital District. In addition, thorough ethical consideration was used when planning and carrying out the interviews. The interviews were conducted with respect and discretion, and the participants were encouraged to contact the researchers if any questions, doubts or inconvenient feelings arose. The data was analyzed so that its reporting does not reveal the identity of the participants.

## Results

### Characteristics of the Participants

Altogether 103 persons with a mean age of 86 years participated in the CAIDE85+ COVID-19 sub-study and answered the questionnaire. The majority of respondents were female (59%) and two thirds were living alone. More than 70% were following the recommendations of the health authorities to avoid physical contacts. Of the participants, 86% had an MMSE score ≥ 25 and 60% had no walking difficulties (Table 1).

Five of the interviewees were men and 10 were women. At the time of the interviews the oldest participant was 93 years old and the youngest was 80. The average age was 86. The background and life situations of the participants varied: nine of the female interviewees and two of the male interviewees lived alone. One female and three of the male interviewees lived with a partner. One female participant had lost her husband during the COVID-19 pandemic. Most of the participants had kin: children, grandchildren, and great-grandchildren. Two of the female interviewees did not have children of their own but had frequent contact with their siblings’ children and grandchildren. All the interviewees were able to live at home without daily help and assistance and were able to go out of the home independently (see also Table 1). All participants lived in urban or suburban environments located in Eastern Finland. However, two of the male interviewees spent several months at their summer cottage located in a remote area.

### Factors Associated With Higher Levels of Meaningfulness

**FIGURE 1:**
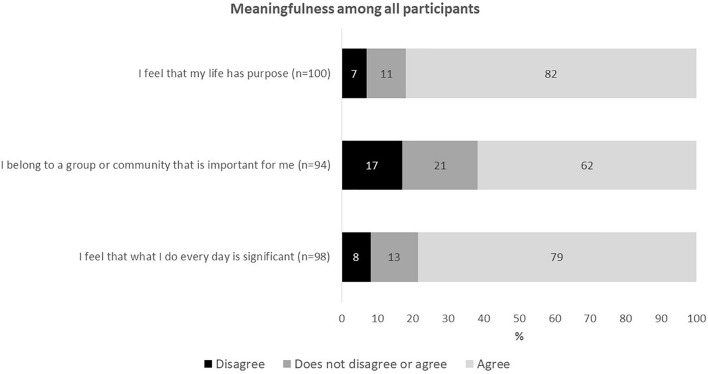
Perceived meaningfulness in the oldest old during the Covid-19 pandemic measured with the Social Inclusion Scale.

A total of 82% of participants felt that their life has purpose and 79% felt that what they do very day is significant. However, only two thirds of participants felt that they belonged to a group or community that is important for them (Figure 1).

**FIGURE 2:**
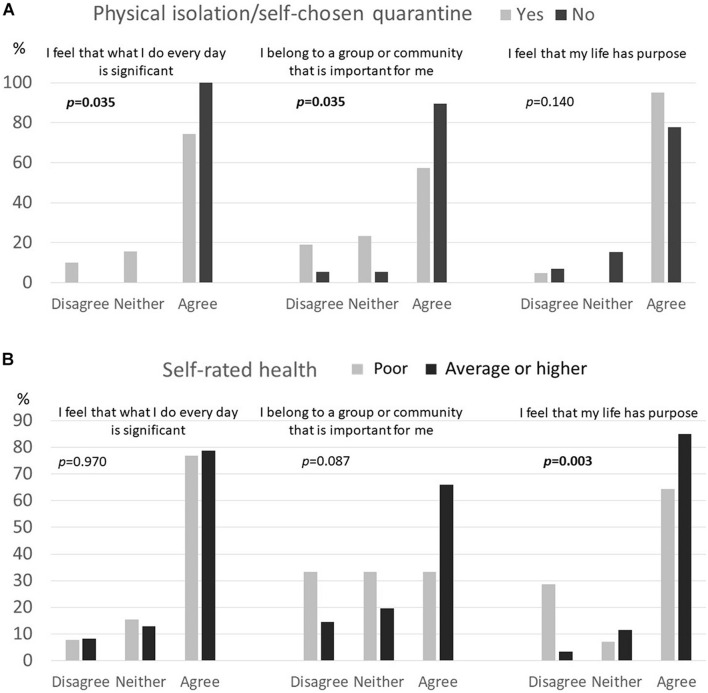
Association of three meaningfulness items with **(A)** physical isolation/self-chosen quarantine status and **(B)** self-rated health among community-dwelling oldest old in Finland. Indicated *p* values analyzed with Chi-square test.

When it was examined which factors were associated with the three meaningfulness items, we found that those with better (at least average) self-rated health felt more often that they have purpose in life as compared to those who reported their health to be poor (*p* = 0.003; Figure 2; Supplementary Table 1). Also those who were not following any physical isolation/quarantine measures felt more often that their everyday life is significant (*p* = 0.035) and that they belong to a group or community important for them (*p* = 0.035) as compared to those who were following some self-chosen isolation/quarantine measures (Figure 2; Supplementary Table 1). We found no evidence for difference between the three meaningfulness items and age, gender, living status, physical functioning or cognitive capacity (Supplementary Table 1).

**FIGURE 3:**
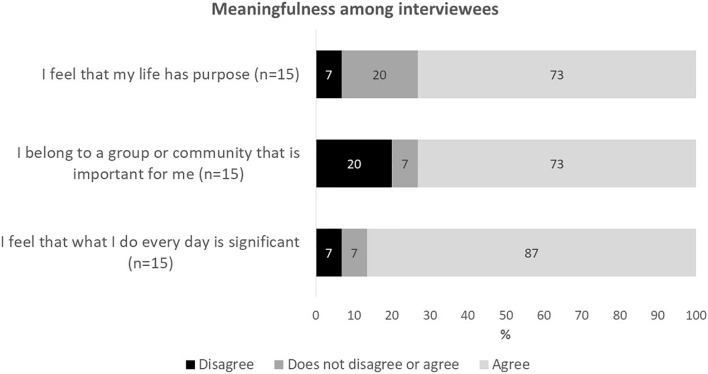
Perceived meaningfulness among respondents participating in qualitative interviews.

In addition to all the study participants, we examined meaningfulness among the interviewees. The majority of them felt that they had purpose in life (73%), that they belonged to a group or community important for them (73%) and also that what they do everyday is significant (87%) (Figure 3). No significant differences were observed between all participants and the interview participants regarding perceived meaningfulness. In the following, we examine the interview participants’ experiences in greater depth by focusing on factors contributing to everyday life meaningfulness during the COVID-19 pandemic.

### Factors Contributing to Everyday Life Meaningfulness

Based on the qualitative analysis, everyday life meaningfulness was supported by the following factors: meaningful social contacts, daily chores and activities, and meaningful places and seasonal changes. The exploration of the sub-themes (Table 2) is interwoven in the analysis of the main themes. Illustrative quotations (in italics) are identified with “F” referring to a female and “M” to a male interviewee, and with a specific number for each participant.

#### Theme 1: Social Contacts

Contacts with family, friends and acquaintances were mentioned several times during the interviews as an important part of daily life during the pandemic. Most of the interviewees kept in touch with other people daily by telephone. Both short and long phone calls were described as something that was anticipated and enjoyed when physical contacts were restricted. “*Phone bills are high this year*,” one of the female participants (F13) stated, laughing. “*We call in turns, whenever something comes to mind. We talk until the batteries run out* [laugh],” described another (F13) when talking about long phone calls with old friends. For one of the interviewees the telephone was the only way to keep in contact with her sister who lived in another city. Due to the pandemic, the sisters had not had a chance to see each other face-to-face, but the relationship was continued through the phone lines:

*Yesterday when we were talking on the phone, and even though we call so many times, we were thinking that every time we talk a new memory comes to mind and then we start discussing. And if we have watched the same television series, we call after that and discuss about what’s going to happen next. Nice to wait and see if we were right*. (F9).

In addition to traditional phone calls, WhatsApp and video calls were also used to keep in touch with family members. Photos sent via WhatsApp, and seeing familiar faces on the screen of a mobile phone or tablet, were seen as meaningful things and ways of keeping the family together. One of the female participants (F2) described using video calls for the first time during the pandemic. Video calls were made with her daughters’ family, who lived further away: “*There’s everyone there and the whole family peeking from the screen, their children also peeking from there*.” “*Then they send loads of WhatsApp photos*,” she added. WhatsApp and video calls were also familiar to the other interviewees. One of the male participants (M3) talked about their family group that was actively used for messaging family members in three generations: “*Even the grandchildren’s spouses are in the group. It’s a way of keeping in contact, it’s something that in a way keeps the whole family together*”.

Video calls were especially important when new family members had been born. “*They have a little boy and I always get a video call from them. It’s in a way nice because otherwise I wouldn’t see how the boy has developed and grown*” (F8). Video calls were also used to keep up with the activities of older grandchildren. These remote contacts with grandchildren were meaningful in many ways:

“*One of my grandchildren is a musician, a music teacher, and he lives in another city, so he has played some corona concerts for us. I have enjoyed them so much, especially the possibility to hear the familiar voice of a loved one*.”(F12) However, the “old fashioned” way of talking on the phone was not a stranger for younger generations too. On the contrary, it seemed to be a meaningful form of communication for both parties, as one of the female participants (F13) described:

“*For example, the night before yesterday, at 10.30 in the evening, one of the grandsons called and said that ‘granny came to mind so I had to call.’ He knows that I’m a ‘late bird’ and sleepy in the mornings* [laughs] *so that he can call even though it’s late. Other grannies sleep but we are still awake*”.

In addition to remote contacts, some of the participants met face-to-face with family members on a regular basis despite the strong recommendations to avoid physical contacts. Children helped with daily chores and groceries, for example. Most importantly, these contacts made the interviewees feel that they were being taken care of. One of the female participants (F9) reported that her daughter just pops by and says: “*I have no business, I just came by to stalk you [laughing]. Is everything all right with you?*”. The interviewee continued describing that: “*Sometimes the kids bring food, and even ask if I have money [laugh]*”, and added that it feels good to know that they care. For another participant (F15), Saturday evenings were particularly important, as her daughter came by to take her to the sauna and wash and oil her back. Together the mother and daughter also cooked a nice meal together.

Meaningful social contacts were also maintained outside the interviewees’ homes. Many of the participants described going for walks with friends and neighbors, or meeting them in the yard. “*Even though I live alone here in a terraced house, we have this little own yard and when the weather got warmer, we started meeting there. It made things easier when you could see and talk to someone face-to-face*,” one of the participants (F5) depicted. Even more, spontaneous contacts with neighbors were seen as nice ways of keeping in contact with others: “*This young couple talks over the fence and last night when the young man started grilling my dog started to bark so I just called out to him that you’ve been spotted. It’s like this in our* [neighborhood] (F2).” One male participant (M10) spoke about having neighbors with whom he could reminisce about the good old times or reflect on current feelings, even though they didn’t know each other that well: “*One day I was cleaning out the yard and one older couple came across the street and the wife went through the gate but the man stayed there and we talked for a long time*”.

For many, neighbors provided not only social contacts but also important support during the strictest time of physical isolation. “*We call them “the shopping bags*,” *the two couples that have helped us with the groceries* […] *They went shopping for us once a week in turns so that it didn’t become too much of a burden for one of them* [laughing].” (F4). The interviewees also described supporting others by offering help for other older persons who had difficulties leaving the home alone. One of the female participants (F2) talked about meeting up with her relative, taking walks together or sitting in the balcony: “*She is in a bit poorer condition than me, she has a lot of illnesses. This one time I said that I will come and pick you up and bring you here and if you can walk back, I will come with you but if you don’t have the strength to walk back, I will take you by car. Twice I’ve done this for her. Once I went to fetch her and we walked back, and then I took her home in the car. And sometimes we’ve just sat on her balcony. Once we’ve sat inside but usually we go for a walk*.” Another participant (F9) described herself as being the one who has been encouraged to go for walks by a friend: “*In the spring time it was nice that one friend always lured me out, saying let’s go for a walk by the lake or do this or that outside*.” Going outside, walking and observing nature also offered meaningful contacts with other people:

“*When you go outside for a walk and bump into someone you know, it’s always nicer than being on your own. And when we sit there by the bench and watch the ducks, it’s not so nice when you are alone, but if someone else is sitting next to you, it’s a lot nicer, even though you don’t even talk to each other*.” (F9).

#### Theme 2: Daily Chores and Activities

Everyday life meaningfulness was associated with having meaningful things to do in and outside one’s own home. For many, familiar daily chores were described as something that supported well-being during the pandemic. One of the male participants (M7) spoke about different chores which he did both at home and at the summer cottage. Time went quickly when having meaningful things to do, such as washing and folding the laundry, and “*sorting out the tools*.” He also worked as a landlord for people renting a summer cottage and enjoyed warming up the sauna for new visitors, as well as taking care of local orienteering routes voluntarily. The interviewee described living “*a modest life*” and spending a lot of time alone, which wasn’t a problem for him when having meaningful things to do. Sometimes daily chores were done with other people, such as his daughter:

“*With my daughter we have picked blueberries and lingonberries. On my own I’ve picked all of the currants. Now I have about 250 liters of berries, in a big freezer at home and twenty liters at the summer cottage. Enough until next summer*”.

Daily chores, such as housekeeping, were also mentioned by several other participants. The last interviews were performed in late November and early December, and Christmas was on the way. One of the female participants (F13) lived with her husband in a senior house, a block of flats with individual apartments. She described social life as being mainly at the house due to the COVID-19 restrictions, and the darker winter season as being difficult because one couldn’t spend time outside so much. Preparing the home for Christmas was something she anticipated and enjoyed even though it would be spent without the wider family: “*Just a week ago we prepared our home in the same way as in the springtime for summer. Now we put some Christmas colors, a few rugs and things like that, so that the place is nice and tidy. There’s a small Christmas tree waiting in the balcony, not decorated yet [laughing] but little by little we’re getting ready for the celebration*”.

Many of the interviewees described receiving help from their children with housekeeping and other chores, but mostly they enjoyed doing them on their own, while they were still able. The interviewee (F15), who spent Saturdays with her daughter going to sauna and cooking, mentioned that it was sometimes irritating that the family always wanted to help with everything. Doing things on your own was an important way of maintaining autonomy in everyday life. Even the smallest chores felt meaningful, which may be especially relevant during a time of restrictions and confinements. “*They always say that they can do it, and I say that let me do these things myself. It keeps me active. At least I feel that I have always been the kind of person who does things and walks al lot, I don’t sit so much. If I don’t have anything to do, the time feels long*”.

Reading, knitting and doing crosswords were mentioned as important activities at home. Due to the lockdown, libraries were closed for several months but the interviewees had found different ways to access new books. One of the participants (F5) told about an arrangement that was made with a her niece’s neighbor: “*This woman has a really big library [in her home]. I got many books by Finnish authors from her which I like to read, and we exchanged so that she brought a bag of books to my steps and took the ones I had read away. We called each other to agree on what books I would take and she had a track on what I had read and what not. It was a really great thing because I read a lot, all sorts of things on different topics*”.

Some of the other participants also had family members or friends bringing them books or crosswords. “*Book service from the son*,” as one of the interviewees (F8) described. “*They ordered me three different crosswords. Making sure that I have tasks to do [laughing]. It [doing crosswords] keeps the mind fresh and maintains brain activity*.” said another participant (F5). Learning new things was seen as an important part of keeping everyday life meaningful, as one of the male participants described when discussing about books and other things that helped avoid boredom:

M11: *After all, there’s a wicked pile of books here, so I can just read them when I have the time. And newspapers are also coming.*

Interviewer: *So, you like to read books a lot?*

M11: *Well, as long as I can and when there’s no other job to do. You have to read; you would get stupid if you didn’t look for wisdom from the books. A person’s learning does not end until their last breath.*

For a few of the female participants, knitting played an important role when staying at home. One of the interviewees (F8) described that her mood was quite low in the beginning of the pandemic and feelings of hopelessness came to mind at times. The summer went better and when autumn came, she started knitting wool socks for Christmas presents and time had gone by nicely. Later on in the interview, she stated that maybe the wool socks saved her:

“*But have these wool socks saved me [laughing], I’ve knitted them so much. I have done long ones, colored ones, many kinds, and it’s given me a nice job to do and I haven’t had time to worry about unnecessary things*”.

Another interviewee (F2) mentioned going to a craft club before the pandemic and when the meetings ended due to the lockdown, the group continued doing hand crafts at home and sharing their activities with each other. Knitting was an important task for her and she made wool socks for her relatives, especially for the younger generations. “*I’ve heard that now there are enough. I have some in storage if someone’s wear out*.” However, meaningful activities did not always stay the same, as described by one participant: “*In the spring time and part of the summer I knitted a lot but then it came to an end, I got fed up of it [laughing]. Now there’s an unfinished sock waiting there patiently in a bag. But for some reason, I now hate to take the needles in my hand*.” (F12).

Going outside for walks was mentioned several times during the interviews, as also reported in the previous section regarding social contacts. Many of the participants described taking long walks and enjoying the outdoors. Walks were a nice way of watching nature and seeing what has been happening in the neighborhood. They were also seen as important in keeping up functional capabilities and monitoring one’s own health, as one of the male participants (M3) described: “*I have a program on my phone that measures the walked distance and so forth and I’ve set a daily target for myself, at least 6000 steps a day so that’s what I try to do. It was a kind of encouragement to move at least that much, even though I’m not a super sportsman. Just to reach the target and satisfy the physical need*”.

Going for walks was especially important for those interviewees who had dogs. It was something that had to be done, but also something that was really enjoyed in the midst of the pandemic. One of the female interviewees (F2) did not have a dog of her own but had an arrangement with her daughter. Already before the pandemic, the daughter had bought a new dog, which was assigned as “*a walker*.” Together they go on walks on a daily basis. The participant picked the dog up from her daughter’s house in the morning and took him back in the afternoon.

“*Since last Autumn we have been for walks. We go along the forest track, our round was four or five kilometers. Now he is sleeping in the swing. We went for a long walk this morning too*”.

Taking the dog out has also provided a chance for other meaningful encounters. Importantly, being outdoors and seeing people there was something that was considered to be a safe thing to do when the virus was still prevalent.

“*There are so many dogs here, and they’re familiar to me from the time my daughter had her previous dog. It’s nice to see familiar faces here [when walking the dog]. We talk, I don’t know all their names, only some of them, but it’s good that you can see each other outside. I think it’s safe and we’ve kept a safe distance*”.

#### Theme 3: Places and Seasonal Changes

The third theme present in the interviews was connected to meaningful places and seasonal changes that were described as supportive factors in everyday life. One male participant (M6) talked about a “camp site” where he had met up with a group of men for over 30 years. The group continued meeting there after the summer, when the COVID-19 incidences had started reducing in Finland. The composition of the group varied over time, but the place stayed the same. Together the men went to the sauna and had an evening snack before heading back home. The organizer of the meetings was a local priest who felt that it was important to keep the meetings going during the pandemic: “*He was brave to open them again, now we just had to sign up for it in advance*.” The same participant visited a local residential house daily when it had opened up after the lockdown. Even though contacts were restricted, and people wore masks, popping by the house and reading newspapers was an important part of his days. The interviewee also had a summer cottage where he headed occasionally: “*Fifteen nights this summer*.” Sometimes alone and sometimes with his lady friend.

The summer cottage played an especially important role for two of the male interviewees who described spending most of their time there in spring, summer and autumn:

“*When we are in the city in the wintertime it’s restrictive, even though there’s more stimulation there. But there’s always a longing to be here* [to the summer cottage] *and as soon as the lake ice melts, we come here. And a bit before the lake freezes, we try and get back [to the city]*”.

In the beginning of the pandemic, the chance to go to summer cottage felt like a big relief:

“*There’s no corona here on the island and not many cottages, people visit them rarely and we are now all alone on the island. It’s like being in in palm of the Lord’s hand here. We have nothing to be worried about here as long as we have something to eat. There’s mushrooms and berries to pick and store, and you get fish from the lake. But we don’t hunt, we buy meat from the store*”.

Other interviewees described the summer cottage as a place with good nature tracks and fishing places, which offered meaningful things to do especially in the summertime. In addition to smaller chores, the summer cottage provided a place for bigger projects too: “*Last time we took down ten big aspens and the next thing is to turn them into firewood*.” one of the male participants (M10) described, adding that life in the city is a lot quieter and less eventful than at the summer cottage. Enjoying nature and the outdoors was also important for those without a summer cottage. Smelling the fresh air and watching nature and wildlife was something that was meaningful in many ways:

“*This summer my sister’s children have taken me out to nature, to sit by a camp fire. So I have had a chance to enjoy nature still. They organize these trips for me because they know that I am a nature person. To listen to the birds singing and to drink coffee that’s been cooked on a fire place*.” (F5).

Even though most of the participants had restricted physical contacts and had taken the national recommendations seriously, all of them felt that it was important to leave the home regularly. Being outdoors was seen as a safe thing to do and importantly, as a way of maintaining well-being during the pandemic. However, it was mentioned that not all older people did the same: “*Some thought that the restrictions included going outside too. I didn’t think so if you walk on your own by foot or by bike. I went outside the whole time; I didn’t stay here inside four walls. I just used my common sense, I can’t get it [the virus] from there [outside] if I don’t meet up with anyone*.” (F5).

As mentioned also within the other themes, spring and summer time played an important role in the participants’ everyday life experiences. Going outdoors and enjoying nature was strongly related to the bright and warmer seasons, which were in many ways anticipated when the pandemic began. “*It was a wonderful experience when spring came after the dreary months*.” one of the participants (F8) described. She had gone for long walks in the springtime and reminisced how it felt when nature started to wake up: “*There’s this old road here and I remember how the birds sang there and the first coltsfoots. It felt like an old poem: the spring still dared to come without asking corona’s permission*”.

The final interviews were made in late 2020, when the darker days were clearly a challenge for some of the participants. Feelings of uncertainty and fear of what was going to happen with the virus was present in some of the interviewee’s minds. However, even in these discussions the interviewees expressed hope for the future. And with seasonal changes, good times were about to come: “*The world doesn’t look good but it’s no use mourning, you just have to wait for spring. After Christmas the days start getting longer, and we will see the sun shining. Then it will be OK again*.” (F13).

## Discussion

Our findings show that despite the changes in daily life due to the COVID-19 pandemic, the oldest old reported relatively high levels of meaningfulness of life. The prevalence was highest in items referring to having a purpose in life and experiencing daily doings as significant. The lowest prevalence was found in the experience of belonging to a meaningful group/community. Within these items, differences were found related to physical isolation and self-rated health. Individuals who were not following any physical isolation measures found more often their daily doings significant and belonging to a group or community important for them as compared to those who followed some physical isolation measures. Individuals who had higher self-rated health (at least average) felt more often that their life has purpose.

The qualitative findings show that everyday life meaningfulness was supported by meaningful social contacts, daily chores and activities, places and seasonal changes. Social contacts were maintained and pursued inside and outside one’s own home. Meaningful daily chores and activities included housekeeping chores, tiding up the yard and doing housework at home or at the summer cottage. These chores kept oneself busy and contributed to one’s sense of autonomy. Everyday life meaningfulness was also supported by meaningful places, such as summer cottages, forest tracks and campfires. Having the opportunity to enjoy nature and outdoor life was closely connected to seasonal changes, which were described as factors impacting well-being in everyday life. Lighter and warmer seasons were something that were looked forward to, and they also brought hope during the pandemic.

Existing research has not examined the oldest old’s experiences of meaningfulness during COVID-19. However, many of our findings are in line with previous studies that have examined older adults’ experiences related to the pandemic. Large-scale survey studies have shown that older people have experienced less negative impacts due the pandemic than younger people (e.g., Birditt et al., 2021; O’Connor et al., 2021), and interview studies that older people have found different ways of coping with the pandemic and maintained optimism toward the future (e.g., Brooke and Clark, 2020). Similarly, our findings indicate that the oldest old have found ways of experiencing everyday life meaningfulness at a time when many familiar and important activities have been compromised. Hence, it may argued that the ability to maintain meaningfulness may be a relatively stable experience in life and not easily affected by changes in health or by global phenomena, such as the pandemic.

Our results show that experiencing meaningfulness does not necessarily require a big effort but is built around small and even simple things in daily life, such as talking on the phone, sitting on a park bench or knitting a wool sock for a loved one. This is in line with existing meaningfulness research, which has emphasized that even “mild experiences” enhancing positive affect can promote a sense of meaning in life (Heintzelman and King, 2014). Factors supporting everyday life meaningfulness, are also closely connected to the sources of joy and comfort, found by Whitehead and Torossian (2021): family/friend relationships, digital social contacts, and hobbies. Regarding the supportive role of hobbies and other daily activities, the researchers suggest that the distraction offered by these types of activities may be particularly adaptive in situations in which stressors are outside one’s control, as in the case of COVID-19.

Interestingly, our findings highlight the importance of autonomy in meaningfulness. Having the opportunity to take charge of your life and even “rebel” against governmental instructions may provide a stronger sense of meaningfulness if recommendations coming from outside are regarded as something that challenge the life worth living. Not following any physical isolation and/or quarantine measures has of course concrete impacts on social relations, which have been regarded as a foundational source of meaningfulness in life (Hicks and King, 2009). People who have kept seeing family and friends despite national restrictions, may have experienced less loneliness and social isolation and therefore felt more meaningfulness (see Heintzelman and King, 2014). Social relationships appear to be especially relevant for older adults with functional disabilities. The relation with meaningfulness and self-rated health may also reflect both disease pathology (Kananen et al., 2021) and the psychological impacts of self-rated health (Han, 2002), which requires further attention in the context of older people’s everyday life.

Previous meaningfulness research has shown that meaningfulness is rooted in fulfillment of human needs, such as finding a purpose and positive self-worth in life. Less focus has been given to the everyday life factors and experiences contributing to these needs, which has been the main aim of our study. Further research is needed to investigate the long-term effects of the pandemic for everyday life meaningfulness of the oldest old, as well as the different factors impacting experiences of meaningfulness in challenging times. Most of our study participants had social contacts and interaction with others, but for many, the pandemic has cut off all social connections. In future research, it will be important to examine the impacts of extreme isolation on everyday life meaningfulness in greater depth.

### Limitations and Reflections of the Study

A possible limitation of this study is the selection of study participants. Even though the CAIDE85+ study is a population-based study, it is probable that the participants who answered the COVID-19 sub-study represent older persons who have better functional capabilities compared to the general population at age 80 and over. As described in the data section, a high number of participants (*N* = 157) declined in participating in COVID-19 sub-study, and for many the reason for declining was poor health and/or poor reserves of strength. It may be that those older persons for whom the pandemic was most challenging did not participate in this study. Another limitation of the study is the relatively low sample size in the survey. Power calculation were not computed for the optimal sample size in the survey. Rather, a general aim was to receive as many survey respondents as possible from the CAIDE population that has been followed up for several decades. In future research, the previous follow-ups may be used to examine changes in meaningfulness, which was not possible with this single data set. Moreover, we cannot completely rule out the possibility that the observed statistically significant differences have been found only by chance.

Limitations in some of the measures used in the quantitative analyses are also acknowledged. The quantitative questions on meaningfulness have been drawn from a scale that has not been validated yet, and that has not been developed to measure meaningfulness *per se*. Thus, caution needs to be taken when interpretating the quantitative results on meaningfulness, as we cannot be sure whether the items tell about meaningfulness or other aspects of the participants lives. However, to our understanding the original Finnish language items may reflect meaningfulness more than the translated ones, which is an often-found challenge in research on meaningfulness and meaningful life (Leontiev, 2006). Regarding other measurements, we note that MMSE is a very crude measure to detect cognitive impairment. However, for the purpose of our analyses, we regard that it sufficiently separates individuals with higher and lower cognitive status.

As a limitation of the study, it is also important to note that e.g., ethnic minorities were not represented in this study. Their experiences may vary from those of other members of the population age group, as findings from Portacolone et al. (2021) have shown. In addition, many of the interview participants represented older adults with good financial and material resources, including a summer cottage, for example. Further research is needed concerning the role of different socio-economic factors in the everyday life meaningfulness of the oldest old.

The timing of the data collection may have had an impact on the findings. The data was collected between July and December 2020. The first surveys were answered in July and the last phone interview was conducted on the 1st of December. This 6-month period can be described as the end of the first wave of the COVID-19 pandemic followed by a period of low numbers of COVID-19 cases and the rise of the second wave of the pandemic in Finland. However, during the 6-month study period, the incidence rate of COVID-19 was considerably lower in Eastern Finland than in many other parts of Finland, which allowed the CAIDE85+ face-to-face main study assessments to be completed between August and December 2020. If the data had been collected at the beginning of the pandemic, the findings may have been different, as many of the participants were in full physical isolation at that time and the virus was something unknown and threatening to all of us (e.g., Brooke and Clark, 2020; Portacolone et al., 2021).

Temporal aspects are present also in the seasonal timings which varied from summer to autumn and winter in our data collection. As shown in the qualitative results, the darker seasons were perceived as more difficult and challenging for well-being. Hence, the survey, which was mostly collected during the summertime, may provide a more positive picture of the oldest old’s experience compared to data collected during the wintertime. However, the mixed-method approach and the rather long period of data collection did provide a nuanced view of the oldest old’s experiences. The variety of seasons may also be seen as strength of the study as seasonal changes play an important role in the everyday life of older adults (e.g., Victor et al., 2015).

### Conclusion and Recommendations

The study gives voice to community-dwelling oldest old’s own experiences during the COVID-19 pandemic, which have received little attention in scientific research but also in the public discussion. As oldest old are the most vulnerable age group in getting a severe or fatal COVID-19 (Hägg et al., 2020; Onder et al., 2020), their experiences warrant special attention. Our findings show that despite protective measures, it is important to ensure that older people have the possibility to maintain self-determination and make decisions regarding one’s everyday life. Moreover, the findings highlight supportive factors in the oldest old’s daily life, as well as their own ways of pursuing meaningfulness in challenging times. Everyday life meaningfulness of the oldest old can be supported in many ways: ensuring social connections, meaningful activities and the possibility to go outdoors and to places important to oneself. In future pandemics, as in “the new normal,” it is important to take into account these different factors and to develop strategies and services enabling older people to live their lives in meaningful ways.

## Data Availability Statement

The data analyzed in this study is subject to the following licenses/restrictions: Access to the data is maintained by the CAIDE research team. Requests to access these datasets should be directed to Mariagnese Barbera, mariagnese.barbera@uef.fi.

## Ethics Statement

The studies involving human participants were reviewed and approved by The Research Ethics Committee of the Northern Savo Hospital District. The patients/participants provided their written informed consent to participate in this study.

## Author Contributions

ET: conceptualization, methodology, investigation, data curation (qualitative), writing—original draft, writing—review and editing, and visualization. IL: conceptualization, methodology, formal analysis, data curation (quantitative), writing—original draft, writing—review and editing, and visualization. EK: data curation (quantitative), writing—original draft, and writing—review and editing. TN: resources, data curation, project administration, and writing—review and editing. AS: resources, data curation, supervision, project administration, funding acquisition, and writing—review and editing. MK: resources, supervision, project administration, funding acquisition, and writing—review and editing. JK: conceptualization, methodology, investigation, resources, writing—original draft, writing—review and editing, visualization, and project administration. All authors contributed to the article and approved the submitted version.

## Conflict of Interest

The authors declare that the research was conducted in the absence of any commercial or financial relationships that could be construed as a potential conflict of interest.

## Publisher’s Note

All claims expressed in this article are solely those of the authors and do not necessarily represent those of their affiliated organizations, or those of the publisher, the editors and the reviewers. Any product that may be evaluated in this article, or claim that may be made by its manufacturer, is not guaranteed or endorsed by the publisher.

## Footnotes

1 Leemann, L., Martelin, T., Koskinen, S., Härkänen, T., and Isola, A.-M. (under review). *Development and Psychometric Evaluation of the Experiences of Social Inclusion Scale*.
