# Supplementary material for: Everyday Life Meaningfulness for the Community-Dwelling Oldest Old During the COVID-19 Pandemic
Source: Front Psychol. 2021 Sep 9;12:716428. doi: 10.3389/fpsyg.2021.716428 (PMC8459014; doi:10.3389/fpsyg.2021.716428)
Supplement: Supplementary file 1 [file Table_1.pdf]

Supplementary Table 1. Associations between three meaningfulness items and sociodemographic factors, health-related factors and physical isolation status among community-dwelling oldest old in Finland during the Covid-19 pandemic.

		I feel that what I do every day is significant				I belong to a group or community that is important for me				I feel that my life has purpose			
		Disagree	Neither	Agree	*p* Value*	Disagree	Neither	Agree	*p* Value*	Disagree	Neither	Agree	*p* Value*
		% (n)				% (n)				% (n)			
Gender	Women	10.3 (6)	10.3 (6)	79.3 (46)		21.1 (12)	14.0 (8)	64.9 (37)		11.7 (7)	10.0 (6)	78.3 (47)	
	Men	5.0 (2)	17.5 (7)	77.5 (31)	0.416	10.8 (4)	32.4 (12)	56.8 (21)	0.075	0.0 (0)	12.5 (5)	87.5 (35)	0.080
Age	<82.5 y	3.2 (1)	16.1 (5)	80.6 (25)		15.6 (5)	28.1 (9)	56.3 (18)		6.1 (2)	6.1 (2)	87.9 (29)	
	85.5–88.8 y	8.6 (3)	11.4 (4)	80.0 (28)		21.2 (7)	15.2 (5)	63.6 (21)		8.6 (3)	11.4 (4)	80.0 (28)	
	<88.8 y	12.5 (4)	12.5 (4)	75.0 (24)	0.728	13.8 (4)	20.7 (6)	65.5 (19)	0.719	6.3 (2)	15.6 (5)	78.1 (25)	0.779
Lives alone	Yes	10.3 (6)	13.8 (8)	75.9 (44)		22.2 (12)	16.7 (9)	61.1 (33)		10.3 (7)	15.5 (9)	74.1 (43)	
	No	8.3 (2)	8.3 (2)	83.3 (20)	0.738	12.0 (3)	28.0 (7)	60.0 (15)	0.363	3.8 (1)	3.8 (1)	92.3 (24)	0.157
Physical isolation	Yes	10.0 (7)	15.7 (11)	74.3 (52)		19.1 (13)	23.5 (16)	57.4 (39)		4.8 (1)	0.0 (0)	95.2 (20)	
	No	0.0 (0)	0.0 (0)	100 (21)	**0.035**	5.3 (1)	5.3 (1)	89.5 (17)	**0.035**	6.9 (5)	15.3 (11)	77.8 (56)	0.140
Self-rated health	Poor	7.7 (1)	15.4 (2)	76.9 (10)		33.3 (4)	33.3 (4)	33.3 (4)		28.6 (4)	7.1 (1)	64.3 (9)	
	Average or higher	8.2 (7)	12.9 (11)	78.8 (67)	0.970	14.6 (12)	19.5 (16)	65.9 (54)	0.087	3.5 (3)	11.6 (10)	84.9 (73)	**0.003**
Physical functioning	Good	7.1 (4)	14.3 (8)	78.6 (44)		12.7 (7)	21.8 (12)	65.5 (36)		5.4 (3)	8.9 (5)	85.7 (48)	
	Moderate	4.0 (1)	20.0 (5)	76.0 (19)		27.3 (6)	22.7 (5)	50.0 (11)		4.0 (1)	20.0 (5)	76.0 (19)	
	Poor	22.2 (2)	0.0 (0)	77.8 (7)	0.314	22.2 (2)	22.2 (2)	55.6 (5)	0.608	22.2 (2)	11.1 (1)	66.7 (6)	0.208
Cognitive capacity	Higher	7.4 (5)	13.2 (9)	79.4 (54)		10.9 (7)	21.9 (14)	67.2 (43)		4.4 (3)	11.8 (8)	83.8 (57)	
	Lower	10.1 (1)	0.0 (0)	90.0 (9)	0.467	27.3 (3)	18.2 (2)	54.5 (6)	0.338	18.2 (2)	9.1 (1)	72.7 (8)	0.219

*Chi-square test. Statistically significant *p* Values (<0.05) are in bold.
